# Delirious Hyperactivity and Agitation in a Young Male Unveiling an Intriguing Underlying Diagnosis: Case Report

**DOI:** 10.5811/cpcem.33483

**Published:** 2025-05-10

**Authors:** Mitch Garey, Joy McLaughlin, Harman Kaur, Jason Graf, Jacklyn Garcia, Megan De Kok, Alexander John Scumpia

**Affiliations:** * HCA FL Aventura Hospital, Department of Emergency Medicine, Florida; † HCA FL Aventura Hospital, Department of Radiology, Florida; ‡ Rocky Vista University College of Osteopathic Medicine, Parker, Colorado; § Lakeside Medical Center, Department of Emergency Medicine, Belle Glade, Florida

**Keywords:** delirious hyperactivity, agitation, basal ganglia, Fahr disease, intracranial calcifications

## Abstract

**Introduction:**

Altered mental status presentations are commonplace in the emergency department (ED), but not all are due to psychiatric etiologies, even if the patient has had a previous psychiatric diagnosis. It is critical to evaluate for organic causes of a patient’s altered presentation. This case highlights the necessity of a broad workup to correctly diagnose an altered patient.

**Case Report:**

A 23-year-old Haitian male with a past medical history of bipolar 1 disorder, seizure disorder, and developmental delay presented to a critical access ED for altered mental status. The patient was given 300 milligrams of ketamine for delirious hyperactivity and agitation by emergency medical services in the field. On physical examination, the patient was in acute respiratory distress, hypoxic, not tolerating secretions, tachycardic, lethargic, and was subsequently intubated for airway protection. Computed tomography (CT) of the brain without contrast was obtained and revealed findings consistent with Fahr disease.

**Conclusion:**

Fahr disease is a rare neurodegenerative condition that causes accumulation of calcium deposits in the basal ganglia as demonstrated on CT.^1^ Phenotypes can be variable, including symptoms such as parkinsonism, chorea, dystonia, cognitive impairment, and ataxia.^2^ This case illustrates the importance of a broad differential diagnosis and emergent medical interventions for emergency physicians practicing in critical access facilities.

## INTRODUCTION

Karl Theodor Fahr first described calcifications of the basal ganglia with associated dementia nearly a century ago, contributing to prior postmortem case descriptions by mid-19^th^ century physicians Delacour and Bamberger.^1^ Fahr disease (FD) is a rare and progressive neurological pathology characterized by idiopathic, bilateral basal ganglia calcifications. It is known to be insidious and degenerative, presenting with extensive phenotypic diversity. Fahr syndrome is differentiated from FD by having an identified secondary cause of calcification and expands the location of calcification beyond the basal ganglia.^2^ To further complicate understanding of these pathologies, close to 30 different names including bilateral strio-pallido-dentate calcinosis and calcinosis nucleurum have historically been used.^2^,^3^ Most recently, primary bilateral brain calcification was introduced as an alternative to FD, encompassing both hereditary and idiopathic etiologies.^1^,^2^

Clinical symptoms of basal ganglia calcification can be vast, encompassing asymptomatic presentations, mood disorders, and extrapyramidal movement disorders. The increasing variety of presenting symptoms in case reports coupled with the interchangeability of terms used to describe intracranial calcification makes FD both an epidemiologic and diagnostic challenge. Bilateral calcification of the basal ganglia identified on computed tomography is considered a diagnostic hallmark; however, it is not specific and estimated to be found in up to 20% of asymptomatic patients over 50 years of age.^4^ Diagnosis of FD should be especially considered in patients with evidence of genetic involvement, which can be autosomal dominant or recessive. Seven distinct genetic mutations have been associated with primary familial brain calcification, four of which are dominant. Concerns continue to arise in using this data, as roughly half of cases lack specific genetic findings.^5^ The case we describe is one of an acute psychiatric presentation that is notable for the young age of the patient and the diagnostic course, which revealed evidence of FD in a critical access rural emergency department (ED).

## CASE REPORT

A 23-year-old Haitian male with a past medical history of bipolar 1 disorder, seizure disorder, and developmental delay presented to a critical access ED for altered mental status. The patient was given 300 milligrams (mg) of ketamine for delirious hyperactivity and agitation by emergency medical services in the field. Aside from the diagnoses, no additional medical history or situational context prior leading to the patient’s altered mental status was available upon the patient’s arrival to the ED; specifically, there were no reports of known recreational drug use or specific concerns regarding a toxicologic exposure. His initial vital signs upon ED arrival were as follows: temperature, 37.2 °Celsius; respiratory rate, 42 breaths per minute; blood pressure, 245/170 millimeters of mercury; heart rate, 144 beats per minute; and oxygen saturation of 72% on 4 liters nasal cannula.

On physical examination the patient was in acute respiratory distress, hypoxic, tachycardic, lethargic, and was not tolerating oral secretions. Pupils were equal, round, and sluggish to light bilaterally. His neurological and psychiatric examinations were limited secondary to his clinical presentation. The patient was not moving his extremities nor tolerating oral secretions and was immediately intubated via video laryngoscopy with rapid sequence intubation (etomidate, rocuronium, respectively). Propofol and fentanyl were used for sedation as well as a nicardipine drip for blood pressure control and levetiracetam for seizure prophylaxis.

The patient received an extensive workup including comprehensive metabolic panel, complete blood count with differential, arterial blood gas, lactic acid, blood cultures, coagulation profiles, type and screen, urine analysis, creatine phosphokinase, drug screen, salicylate, acetaminophen, and ethanol levels, and influenza and coronavirus disease 2019 testing. Laboratory analysis was significant for hyperglycemia at 172 mg/deciliter (dL) (reference range: 70–100 mg/dL), hypokalemia at 2.9 milliequivalents per liter (mEq/L) (3.5–5.0 mEq/L), elevated creatinine at 1.86 mg/dL (0.7–1.3mg/dL), hypocalcemia at <5 mg/dL (9–10.5mg/dL), elevated lactic acid at 3.9 millimoles (mmol)/L (0.67–1.8 mmol/L), elevated creatine phosphokinase at 2,284 units (U)/L (30–170 U/L), and a positive COVID-19 test, but no single value was ultimately deemed contributory to the patient’s agitation. All other laboratory studies were grossly unremarkable or within normal limits.

*CPC-EM Capsule*

What do we already know about this clinical entity?

*Fahr disease is a neurodegenerative disorder causing basal ganglia calcifications, with diverse symptoms including movement disorders and cognitive decline*.

What makes this presentation of disease reportable?

*The young age at presentation, severe psychiatric symptoms, and discovery in a critical access ED highlight the need for a broad differential diagnosis*.

What is the major learning point?

*A thorough workup is essential for altered mental status to uncover rare conditions such as Fahr disease, which may mimic psychiatric disorders*.

How might this improve emergency medicine practice?

*Early imaging and broad diagnostic considerations can enhance detection of organic causes in psychiatric presentations, leading to better patient outcomes*.

Electrocardiogram, chest radiography, and computed tomography (CT) angiography of the chest were obtained and revealed a right lower lobe pneumonia but were negative for other pathology such as pulmonary embolus or aortic dissection. In addition to the medications, the patient was given piperacillin/tazobactam and vancomycin to cover empirically for aspiration pneumonia and/or sepsis. A 2-liter bolus of lactated Ringer solution was also administered. Computed tomography without contrast of the head was obtained (Image), which revealed findings consistent with FD.

Of note, upon discussion with the patient’s mother, who arrived in the ED during the patient’s initial workup and stabilization, the patient had never previously received CT imaging of the brain prior to this presentation, despite multiple prior presentations of agitation that were attributed to psychiatric disorders. Additionally, the patient’s father presented to the ED with the patient’s mother and stated he had Parkinson disease. The patient’s father was noted at that time to exhibit gross neurologic deficits atypical of Parkinson disease at the father’s age.

The patient was ultimately stabilized and transferred to a tertiary-care hospital where he underwent further diagnostics and management by a multidisciplinary team including an intensivist, a neurologist, and a medical geneticist. Laboratory analysis at the tertiary-care hospital revealed a normal parathyroid hormone level. The patient was found to have a genetic cause of his FD. Both the patient and his father had further genetic testing performed, which revealed an autosomal dominant trait in both, confirming our initial diagnosis of FD.

## DISCUSSION

While adult-onset FD classically presents in the fourth and fifth decades of life, this case demonstrates clinical manifestations in a 23-year-old, who began showing symptoms of cognitive impairment in his early teens. Jaworski et al delineate sub-types of Fahr presentations by age of onset, with childhood onset characterized by developmental delay, early onset (third decade) characterized by psychiatric symptoms, and late onset (fifth decade) characterized by progressive dementia and movement disorders.^5^ Fahr disease has previously been reported to demonstrate an anticipatory genetic effect, wherein subsequent affected generations present symptoms at progressively younger ages, often with increased severity.^6^,^12^ The severity of Fahr symptoms has been correlated to the extent of intracranial calcification, highlighting the importance of early diagnosis and prompt management, especially when due to secondary causes with distinct treatments.^5^

Our patient’s comorbid diagnoses of severe developmental delay, mood disorder, and seizure disorder likely contributed to his delayed diagnosis of basal ganglia calcification. Naqvi et al recently described a case of bilateral calcification of the basal ganglia in a 21-year-old male discovered on CT during evaluation of acute exacerbation of schizophrenia.^9^ That patient had been diagnosed with schizophrenia three years prior and only received CT imaging after having a seizure.^9^ Nearly 40% of FD patients present with only psychiatric symptoms, making the diagnosis challenging.^8^ The utility of imaging is exemplified by Nicolas et al who describe a case in which a patient presenting with psychosis in the fourth decade of life was found to have basal ganglia calcification on imaging.^10^ In that case, the the CTs of both the patient’s parents revealed similar calcifications; however, both of her parents were asymptomatic.^10^

Our patient’s severe hypocalcemia and calcified basal ganglia, as well as his psychiatric and seizure history are highly suggestive of FD. His age and mood symptoms align with bipolar disorder; however, a more thorough evaluation should have included organic causes of neuropsychiatric symptoms, especially considering his seizure history and developmental delay. Additionally, the patient’s father also displayed signs of a neurocognitive pathology, which further suggested a genetic factor. Early diagnosis of this disorder has implications for management and, importantly, for genetic counseling. Cassamina et al described a case of FD with bipolar disorder refractory to pharmaceutical intervention in which electroconvulsive therapy provided complete resolution of mood, cognitive, and behavioral symptoms for several years.^11^

Limitations in this case largely result from the patient arriving in acute respiratory distress.

While intramuscular ketamine is a known sedative agent in acute psychiatric agitation, the patient’s presentation was not consistent with respiratory depression but rather severely altered mental status resulting in the inability to protect his airway. Additionally, the patient’s known history of seizure disorder, which may have caused this patient to present postictal, could have explained his initial symptoms. Moreover, genetic studies would have been beneficial, although the acuity of the patient’s deterioration upon arrival to the ED limited such analyses. Ultimately, it is impossible to conclude definitively what caused this patient’s altered mental status before and upon arrival to the ED. Despite this, the intended objective of bringing this case to light is not to retrospectively explain one event of altered mental status but rather demonstrate the need for a broad differential in such cases, as this patient had a profound and previously unrecognized pathology that had affected him throughout his life.

## CONCLUSION

For patients with newly diagnosed psychiatric disorders accompanied by neurologic symptoms such as seizure activity and/or family history of psychiatric or neurologic deterioration, imaging is a vital element of the diagnostic workup. In this report we did not aim to challenge the current multifactorial understanding of psychiatric disorders but rather to highlight calls for increased use of imaging of new psychiatric and neurologic diagnoses to better characterize the highly variable presentations associated with intracranial calcification. Treatment course varies depending on underlying etiology, which makes early diagnosis vitally important for optimizing patient outcomes.

The genetic anticipation of Fahr disease further illustrates this point, as early genetic counseling can be provided. Further research and systematic review of case presentations are needed to better characterize FD and cement the naming scheme of the pathology. Distinct classification of symptoms, quantitative imaging findings, and genetic analyses can create definitive diagnostic criteria, improving time to diagnosis and thus intervention for these patients.

## Figures and Tables

**Image:**
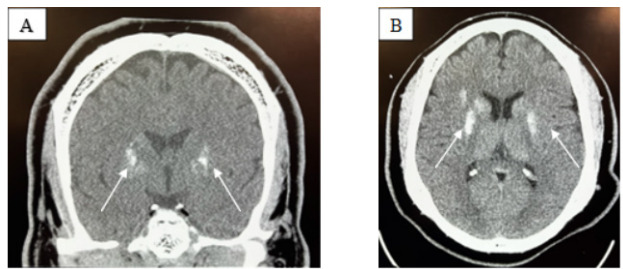
Non-contrast computed tomography of the head: (A) coronal view, and (B) axial view, demonstrating bilateral basal ganglia calcifications (white arrows) in a patient with Fahr disease.
